# Preface

**DOI:** 10.3897/zookeys.147.2162

**Published:** 2011-11-16

**Authors:** Robert L. Davidson

Ross T. Bell: all of us in the group photo of this Festschrift are celebrating his 80^th^ birthday and his long and industrious career with Joyce Bell, his wife, and most times co-author.

What can you say about a man who could reel off the catalogue of Popes from Peter to present, could recite the list of English monarchs from 1066 to Elizabeth II (and many pre-Norman ones as well), and was well versed in the world’s most obscure religions, and could even make jokes in pidgin? You could say that there was never a dull moment when hiking the Green Mountains or camping overnight in some ramshackle lean-to on Camel’s Hump, not to mention when sucking down a gin and tonic back home. And complemented by Joyce Bell, who was no slouch when it came to knowledge of the Vermont flora and fauna, enlightenment and enjoyment were profuse during the many field adventures undertaken over the years with me and myriad other students and colleagues of the Bells.

When George Ball first approached me about planning a BellFest similar to the BallFest we had done in 2006 when George turned 80, he thought it would be fairly easy to do in conjunction with a meeting of the Entomological Society of America. This would be to honor the Bell’s six decades or so of scientific contributions, and, incidentally, a slightly belated celebration of Ross Bell’s 80^th^ birthday. My response in essence was “great idea” but, alas, Ross can no longer travel very well, or very far. What about trying to bring the celebration directly to Ross and Joyce? This would be considerably more difficult, as we would have to plan the local logistics from several far-flung cities. But ultimately we decided to give it a go.

So we conjured an organizing committee consisting of George, John Spence, Jessica Rykken and me. We gradually developed a plan and a time scheme. I dredged up a number of current and prehistoric names from my correspondence files, as did the others, and George sent out a prospectus and invitations. The early response was absolutely positive.

Unfortunately, conflicting events in Burlington, Vermont, and the lack of a native guide, as none of us lived in Burlington, made it difficult to settle a date as early as we would have liked, and there was also the difficulty of handling from afar local arrangements for lodging, banquet, and a venue for speakers. The brunt of this was borne by John Spence, who rose magnificently to the challenge and eventually overcame all difficulties. John set up a website; I contributed a bibliography; Jessica took over duties as treasurer; and John negotiated with the University of Vermont for lodging, catering for the reception and banquet, and a hall to accommodate the speakers and audience.

We tried to include a broad spectrum of carabid and beetle colleagues, former students, naturalists with a core interest in New England, members of various northeastern entomological societies, university colleagues, and even a few old friends. An impressive number of these was able to make it in spite of the location being a bit off the beaten path and not associated with a larger concurrent meeting. And so it came to pass that on June 12th, 2010, a motley assemblage of professors and teachers, taxonomists and systematists, professional and amateur naturalists converged on Burlington, Vermont, to honor the Bells (see group photo with names). The Bells had spent much of their lives encouraging such motleyness, so it was only fair that we should have our shot at retribution.

An introductory reception took place the first evening, followed by two days of speakers, and on the fourth day a field trip. There were twenty-five presentations, and perhaps sixty participants present in total through the various talks and the field trip. A banquet was held on the second evening, and the field trip was hosted by members of the Vermont Entomological Society, an energetic group that also supplied a nice lunch. As one might expect for the Bells, carabids dominated the talks, but with a liberal sprinkling of ecological papers, and even a few papers on moths, flies and earthworms.

Before the last echo of the last speaker had died away, George had already drafted Terry Erwin to edit a Festschrift for the Bells, and it was decided that ZooKeys would be the venue. During the ensuing year and a few months, Terry took on this thankless, yet rewarding task with great energy and good grace. Most of the papers presented during the symposium were made ready for publication, along with quite a few new submissions from some who could not make the festivities, yet wanted to participate. The results of all this you now have in your hands, so let the games begin ... glasses raised, to the Bells, may long live the Bells–certainly their contributions to science and students and local biotic lore will!

Robert L. Davidson

